# Perspectives of community and facility stakeholders on community health workers in rural Malawi

**DOI:** 10.4102/phcfm.v16i1.4199

**Published:** 2024-02-12

**Authors:** Myness K. Ndambo, Moses B. Aron, Henry Makungwa, Fabien Munyaneza, Basimenye Nhlema, Emilia Connolly

**Affiliations:** 1 Department of Community Health, Partners in Health, Neno, Malawi; 2 Department of Monitoring and Evaluation, Partners in Health, Neno, Malawi; 3 Department of Clinical Services, Partners in Health, Neno, Malawi

**Keywords:** community health workers (CHWs), community-based primary health care, community members, health facility, male involvement, enablers, challenges, Malawi

## Abstract

**Background:**

Community health workers (CHWs) bridge the primary health care (PHC) system and communities by providing care in the household. In Malawi, few studies have examined the perspective of users of household-level CHW services, in remote areas, to understand CHW’s role in community-based PHC.

**Aim:**

To explore perspectives of community and facility stakeholders on the enablers and challenges of the CHW role in community-based PHC in Neno District.

**Setting:**

The study was conducted in the Neno District health facilities, namely, Ligowe, Dambe, Chifunga, and Zalewa.

**Methods:**

We conducted eight focus group discussions (FGDs) with purposively sampled community members and conveniently sampled facility stakeholders. Data were transcribed and analysed thematically through an adapted COM-B model of behaviour change.

**Results:**

Three main themes of perceived behaviour change within the CHW role were identified: (1) *capacity* – the CHW programme aids health education and promotion within the community; (2) *opportunity* – the CHW programme facilitates community-based PHC and linkage to the facility; and (3) *motivation* – the CHW programme enablers and challenges in providing community-based PHC.

**Conclusion:**

Community health workers enrich community-based PHC delivery through health education, timely access to care, and linking communities to the facility. Optimising workload and programme support is critical for the help of CHWs. Further studies are required to address programme and cultural challenges to enhance positive health-seeking behaviours.

**Contribution:**

This study provides contextual knowledge for further research on bringing together spiritual and formal health practices and considering the cultural background when planning for health interventions in remote areas.

## Introduction

Community health workers (CHWs) significantly deliver community-based primary health care (PHC) worldwide.^1,2^ According to the World Health Organization (WHO), CHWs are lay health care workers chosen from their communities to support the local healthcare system in concert with their communities.^3^ Community health worker roles range from patient support to direct care delivery in specific contexts and settings, but still, all are in the influential intermediary position with proximity to households and linking to the facility-based PHC system.^4^ Globally, CHWs contribute to PHC through their roles in the village or household health education and promotion, early identification of disease and linkage to care, providing psychosocial support, and decreasing the workload on primary health facilities.^5,6^

Primary health care services in low- and middle-income countries (LMICs) are subject to staff shortages, a lack of space, and equipment to provide care and stock-outs of medication, often leading to poor quality care delivery.^7^ Despite all these vital components at primary facilities, hard-to-reach and rural communities are usually limited to health care access by geographical distance, rugged terrain, and a lack of transport. Community health workers can provide health education and promotion, screening services, treatment and psychosocial support, and even direct health care access by reaching community members in their homes.^2,8^ Literature demonstrates that CHWs can improve access to health care, decrease social barriers, and provide health education to individuals who may not have previously engaged with available health services.^9^ Through their proximity to households and role as trusted community members, CHWs can detect diseases earlier, link clients to the necessary care, and provide direct follow-up for medication adherence and monitoring.^1^

The CHW role introduces task-sharing with facility-based primary care workers, which decreases the workload for facility staff and strategically places CHWs as an extension of primary care providers and a trusted community member.^1,10,11^ Several studies have demonstrated that CHWs create trusting and linkage relationships with rural communities and facility-based primary care workers.^1,12,13^ Community health workers have been shown to improve health knowledge and access to facility-based care. To perform these functions,^1,12,13^ CHWs require deep community involvement and positive engagement with facility-based primary care workers to maximise their role and competently perform tasks.^14,15,16,17^ Literature has demonstrated that CHWs are motivated by praise and trust from their facility-based colleagues and benefactors, monetary and other economic benefits.^1,17,18^ Despite the community health worker playing a facilitating role, barriers have been reported to community-based PHC provision, including insufficient training for CHWs, supervision challenges, a lack of resources, inadequate confidentiality and community involvement, and poor motivation.^18,19^

While there are multiple studies from the CHW perspective, on enablers and challenges of their role,^1,2,15^ there are fewer on the view of community members^6,8,18^ and facility-based primary care workers.^20^ In Malawi, community health care is provided through the Health Surveillance Assistants (HSA), who support village clinics household-level care, and facility-based tuberculosis (TB) and malnutrition programmes with many additional responsibilities through task-sharing from other health-facility staff.^20^ Unfortunately, HSAs are significantly understaffed. In the Neno District, the current ratio is 1 HSA to 2300 people which is much lower than the expected 1:1000 ratio, leading to a lack of community care delivery.^20^ Most CHW studies in Malawi have focussed on the community HSA who have demonstrated barriers to care.^13,15^ The CHWs in this study provide another layer of care directly at the household level as a helper ‘foot soldier’ cadre to the HSAs aligned with the national CHW structure.

No studies in rural Malawi have focused on community and facility-based health care worker perspectives of community-based CHWs. Through programme implementation and evaluation, we explored the perceptions of community members and facility-based primary care workers on the CHWs’ enablers and challenges in providing community-based PHC in the rural Neno District, Malawi.

## Research methods and design

### Setting

The Neno District is a rural area in southwestern Malawi covering 1469 km^2^ with an estimated population of 150 211 people in 2022.^21^ Neno is one of the poorest and hardest-to-reach districts in the country, with no paved roads to the majority of health facilities, only 3.7% of people having access to electricity, and the majority of the population surviving on subsistence farming for food and income.^22^ There are 15 health care facilities in each of the population catchment areas in the Neno district, with 13 primary health facilities and 2 hospitals, with most of the population still living greater than five kilometres from a primary health facility.^22^

### Programme description

Partners In Health (PIH), locally known as Abwenzi Pa Za Umoyo (APZU), has supported the Ministry of Health (MoH) in the Neno district to strengthen the rural health care system since 2007. In 2007, PIH or APZU and the MoH developed a community-level CHW cadre to provide treatment support to human immunodeficiency virus (HIV) and TB patients in assistance to the HSAs. However, 2016, recognising the need to address the broader scope of the disease burden with integrated community care and sustained behaviour change among all clients, the MoH and APZU or PIH restructured the CHW programme to cover eight priority disease areas – HIV, TB, non-communicable disease (NCD), maternal and neonatal health, sexually transmitted infection (STI), family planning, child health, and malnutrition.

The CHW programme below the HSA is split into three levels. Each household-level CHW is assigned 20–40 households within their community serving as the first level. Community health workers are expected to visit each home at least once a month for health education, screening, referral, accompaniment to the health facility, and psychosocial support. More frequent follow-ups are indicated for patient monitoring, including adherence and medication side effects, and tracking patients with missed appointment visits, especially for HIV, TB, and maternal and neonatal care. At the village level, CHWs collect data on each household visit, which is collated monthly. On the second level senior community health workers (SCHWs) supervise about 10–15 CHWs and are assigned as a primary CHW to 15 households. Senior community health workers have an additional task of community-level TB sputum collection with delivery to the facility for diagnosis. At the facility level, site supervisors (SSs) and HSAs serve as community health programme leaders, receive patients linked by CHWs from the community, and facilitate patient follow-up. The HSAs work hand in hand with SSs to coordinate the follow-up of patients, assign tasks to CHW and SCHW, and run village clinics. The SSs supervise and mentor SCHWs and CHWs in one catchment area at the health facility, and are responsible for catchment-level reporting to support the HSAs at the health facility.^23^

Community health workers are nominated by their communities for the role and are required to have the ability to read and write. The nominated members are interviewed and selected using an aptitude test, and are trained and supervised by SCHW, SS, and HSAs. The population size determines the number of CHWs required in a particular catchment area. Initial training includes a 5-day course with follow-up quarterly 1-day refresher training at each catchment area with quarterly supervision by SCHW and SS.^23^ Community health workers are equipped with job cards, registers, and logbooks for data collection and receive a volunteer stipend.

### Study design and sampling

We conducted an exploratory qualitative study in the phenomenological tradition^24,25^ with data utilising eight focus group discussions (FGDs) between October 2018 and March 2020 with 92 participants including community members and PHC workers in four purposively^26^ selected catchment areas in Neno district: Ligowe, Dambe, Chifunga, and Zalewa. We chose these study sites as they were the first CHWs to visit and screen each household within the eight disease focus areas since 2016.

Community participants were purposely sampled^26^ depending on their proximity to the health facility to ensure that individuals who lived far and near it were included. Facility health care workers were sampled at each included facility, including clinicians, medical attendants, nurses, clerks, lab technicians, site supervisors, HSAs, and other service providers in the targeted health facilities. Participants at health facilities were recruited using convenience sampling,^27^ regardless of age or sex, as the total did not exceed the required number of participants per FGD facility. The number of FGDs was pre-selected because it was more than the minimum number usually required to reach saturation, a point in qualitative research where data collection no longer offers new data.^28,29,30^ Previous studies have shown that four FGDs,^29^ five FGDs,^31^ or six FGDs^32^ are enough to reach saturation. Using diverse samples and inductive coding, saturation was reached with six FGDs, and two more FGDs were added to fully understand the concepts.^29^

### Data collection

In October 2018, four FGDs were conducted with community members in the four catchment areas. In March 2020, one FGD was conducted with primary care workers at the four sampled health facilities in the catchment areas. The study employed FGDs to gain more insights into participants’ perceptions of the CHWs. The focus groups had 11 to 12 participants.

This was part of a more extensive 3-year evaluation of the CHW programme. During the study, APZU made no significant changes to the household model in application or performance. Data collection included: (1) year one data collection, which included community members and CHWs, (2) year two was for chronic disease patients, and (3) year three was for facility-based primary care workers. Qualitative data for this study are taken from years one and three. Data were collected using two FGD question guides developed in English and translated into the local language (Chichewa) for the facility-based primary care workers and community members (Appendix 1). These question guides were pretested among 10 community members and 10 facility-based health care workers at Neno District Hospital. The preseting results were not incorporated in the findings, but assisted in refining the questions for clarity and incorporating additional probes. The participants were asked to describe their perceptions of the enablers and challenges of the CHW role in providing community-based PHC through the expanded, household-assigned model. Question guides included questions about CHW services, acceptability of specific disease focus areas, and competency of CHWs. Further questions were asked about perceptions of CHW trust, influence on healthcare-seeking behaviours, perceived differences between CHWs and other health cadres, gender perceptions, and areas of programme improvement.

Data were collected by the research fellow and three trained research assistants who were well-versed in qualitative research but had no part in the design and implementation of the evaluated project. All FGD participants were identified using pseudo names and were asked to mention the pseudo name before contributing during the FGD. All FGDs were conducted in a private setting as selected by the participants. After every FGD, the research fellow summarised the key findings and shared them with the participants for member checking.^33^ The FGDs took approximately 1 h and 30 min, and we provided travel reimbursements to participants. The discussions were conducted in Chichewa and recorded. Research assistants took notes to supplement the recordings, and the research team debriefed after every FGD.

### Data analysis

Recordings were transcribed verbatim in the local language of Chichewa and then translated into English by the research fellow. Two research team members listened to all recordings and cross-checked the verbatim transcription and translation. The research fellow developed a codebook by inductively examining codes related to general perceptions of CHWs, perception of CHW roles, and resources used by CHWs to facilitate care in the community concurrently with data collection. Two independent investigators checked the codebook for validation by independently reading the first three transcripts line by line and identifying emerging codes to ensure the reliability of coding and consistency. The investigators and research fellow regrouped for a final codebook through a consensus process by looking at commonalities and differences.^34^

The final codebook was agreed upon by the joint consensus of all authors.^34,35^ Then the research fellow coded all transcripts in Dedoose version 8.3.17 using the validated codebook and exported the excerpts to a Word document for easy immersion and/or familiarisation with the data through repeated and active reading.^36^ Finally, all the authors regrouped again and identified relationships between these codes, frequently identified codes were merged, and themes were generated from these codes. We utilised an inductive approach to identify emerging themes of perceived enablers and challenges of the CHW role.

Behaviour change is a critical pillar in the development of the CHW programme in Neno district with the CHWs providing health promotion, prevention and education, and disease screening and treatment monitoring. Utilising the programmatic focus of behaviour change to interpret the qualitative findings, we adapted the COM-B model.^37^ The COM-B model is a theoretical framework which provides an understanding of the behaviour of an individual, group or community through changing one or more of the following components: capability (‘the what’), opportunity (‘the how’), and motivation (‘the why’). Behaviour change is an interaction between one’s capability to perform a behaviour and the opportunity and motivation to carry out that behaviour. A new behaviour or behavioural change requires a change in one or more of these components. A change in *capability* can be physical or psychological; a change in *opportunity* from a change in physical or social environment can facilitate or create barriers to behaviour change, and a change in *motivation* can be automatic (unconscious response) and reflective (conscious response). In this study, we utilise this framework to guide the interactions between *capability, opportunity*, and *motivation* of the CHW programme in community-based PHC delivery. This article consists of summaries, interpretations, and quotes from the exported excerpts.

### Ethical considerations

This study was approved by the National Health Science Research Committee (NHSRC) in Malawi with protocol number 1059, titled ‘Lessons learned from monitoring and evaluation of Community Health Initiatives in Neno district, Malawi’. Written informed consent was obtained from all the participants before data collection. Confidentiality and anonymity were maintained through allocating pseudonyms or numbers to the participants and transcripts. The information letter informed participants of their choice to participate and the option to withdraw at any stage of the research process. This study was conducted as per the Declaration of Helsinki guidelines and regulations.^38^

### Reflexivity

The research assistants and M.K.N. had no prior involvement with the design and implementation of the CHW household model programme. M.K.N. worked with the CHW programme as a research fellow and was hired to evaluate the project. B.N. was the Community Health Director and H.M. was the programme manager. M.B.A. worked as the Monitoring and Evaluation officer during this time. E.C. and F.M. were not involved in the day-to-day monitoring and overseeing of the programme. However, the authors acknowledge their preconceptions and contextual experience in the household model programme, data collection, and analysis that may influence how data were analysed and coded.^39^ Such experiences shaped how the authors analysed and coded the data set. As they reflected on their experiences, they realised that the world is perceived differently from others, and that reality is subjective and multiple. Therefore, drawing on an interpretive framework adapted from the COM-B model, the constructs helped them to focus the direction to highlight and explicate the depth and richness of experiences of both the researchers and respondents as regards the improvement of the community-based PHC.^40^ In addition, our hands-on experience in qualitative research shaped our view of the data and that is the angle from which we position our findings.

## Results

Participant socio-demographics indicate that there were almost equal numbers of community members (49%) and PHC workers (51%) who participated in the FGDs (Table 1). Most participants were aged between 25 and 39 years with greater female representation (60%) from community members as compared to the PHC workers which had greater male representation (60%).

**TABLE 1:** Socio-demographic characteristics of focus group participant categories.

Variable	Community Members		Facility-based primary care workers	
	*N*	%	*N*	%
Number of participants in each category	45	49	47	51
**Location**				
Ligowe	11	24	11	23
Dambe	11	24	12	26
Chifunga	11	24	12	26
Zalewa	12	27	12	26
**Age (in years)**				
12–24	2	4	3	6
25–39	20	44	32	68
40–59	17	38	12	26
60 and above	6	13	0	0
**Sex**				
Male	18	40	28	60
Female	27	60	19	40
**Community members**				
General	20	44	-	-
Leaders†	13	29	-	-
Community-based organisations‡	12	27	-	-
**Facility based primary care workers**				
Clinicians	-	-	8	17
Nurses and midwives	-	-	7	15
Facility CHWs§	-	-	8	17
Ancillary staff¶	-	-	16	34
Non-medical staff††	-	-	8	17

CHW, community health workers.

† , Included chiefs and village headmen;

‡ , Included community-based organisation and village development committee members;

§ , Included health surveillance assistants (HSAs) and site supervisors (SS);

¶ , Included expert clients, health attendants, laboratory staff, counsellors, and pharmacy staff;

†† , Included data clerks, ground labour, and security guards.

**FIGURE 1:**
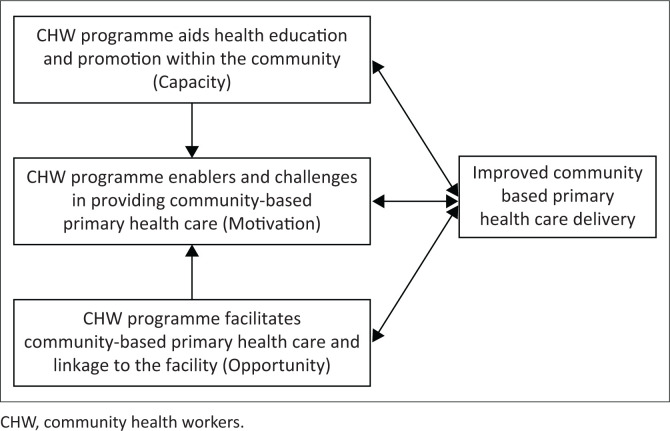
Results interpretation framework adapted from the COM-B model.

Through an inductive approach, emerging themes of perceived enablers and challenges of the CHW role were identified with adapting the COM-B model framework to understand how the CHW programme induced behaviour change for individuals and community-based PHC delivery. Figure 1 shows the mapping of inductive themes to the COM-B model.^37^ The themes include: (1) *Capacity* – the CHW programme aids health education and promotion within the community, (2) *Opportunity* – the CHW programme facilitates community-based PHC and linkage to the facility, (3) *Motivation* – the CHW programme enablers and challenges in providing community-based PHC.

### Capacity (‘the what’): The community health worker programme aids health education and promotion within the community

Participants reported that CHWs help increase health knowledge that decreases stigma and discrimination through household visits in each household in the community regardless of their health status. Community health workers provide health education and promotion for each community member, not only those with specific diseases (i.e. HIV and TB), that enhances individual and community-level understanding of illness and wellbeing:

‘Previously, people were discriminating [*against*] each other and being stigmatised, but with the messages CHWs have disseminated during home visits, people have accepted that everyone can get sick…’ (P2, male, healthcare provider, facility 1)

‘At first, only those that are sick were benefiting, but now everyone in the family is benefiting, and the relationship is very good since the CHW is now linked to all of us… I will reach 100 years now.’ (P6, female, community member, facility 3)

Participants shared that by providing health education, CHWs increased their capacity to prevent disease and promote health by understanding the importance of seeking care for disease symptoms. Participants shared examples where CHWs provided knowledge on how to practise healthy behaviours:

‘Previously, we were washing hands using the same water when eating. CHWs have taught us to pour water on each other, and now there is a huge change in the community.’ (P11, female, community member, facility 1)

‘I used to consult traditional doctors thinking that I was being bewitched, but my condition was getting worse until the CHW visited me and taught me about HIV and AIDS … I am now strong like anyone else. Without the CHW… I could have been dead by now…’ (P11, male, community member, facility 2)

On the other hand, participants additionally reported challenges in the lack of CHW capacity, need for additional training, and the lack of resources to perform all tasks. They felt these deficiencies led to poorer care delivery and miscommunication with clients and the facility-based primary care workers. For instance, health care providers complained that some CHWs confuse clients by telling them the medication that they should receive at the hospital even before the clinician’s prescription:

‘Some CHWs struggle to read. Sometimes they even rely on us to tell them the readings when we have come with children for under five clinics…’ (P4, female, community member, facility 2)

‘CHWs should give information that they are conversant with. That which is within their capacity… they tell clients the kind of medicine they are going to receive at the hospital so if you prescribe differently, you are hated by the clients.’ (P1, male, healthcare provider, facility 2)

‘Insufficient working materials by the CHWs in the communities. For instance charts. Some have while others do not have these materials.’ (P11, female, healthcare provider, facility 1)

Despite the reported gaps in training and teaching resources, community members still appreciate CHW’s ability to provide knowledge in health behaviour change. One community member gave an example of CHWs encouraging individuals to create their own nutritional solutions and resilience when the medical supplements are not enough or stocked out:

‘CHWs also encourage us to eat balanced diet food using our locally available food. For instance, my child was malnourished and porridge supplements were not enough at the hospital. My CHW told me to use locally available foods like making groundnuts flour and also make a home garden of vegetables to make sure that my home has enough supply of vegetables. As I am speaking, my child is back to normal. I am now teaching my neighbours to stop relying on free porridge from the hospital.’ (P7, female, community member, facility 3)

### Opportunity (‘the how’): The community health worker programme facilitates community-based primary health care and linkage to the facility

Participants conveyed that CHWs were essential human resources in facilitating patient access to screening, linkage to facility-based PHC, and treatment follow-up. For example, CHWs conduct TB sputum collection, delivery to the facility for testing and then follow up and report results to community members which improves TB case finding and encourages care-seeking behaviours if diagnosed:

‘When we talk of community TB screening, they are performing very well in the communities as compared to the performance at the OPD [*outpatient department*] … They do well, and we are proud as a team …’ (P2, male, healthcare provider, facility 1)

‘… [*S*]ometimes we just stay here without knowing we are sick. For example, my husband… The CHW came and collected his sputum, and it was discovered that my husband has TB …Without the CHW coming here, I cannot even imagine what could have happened.’ (P8, female, Community member, facility 4)

Similarly, participants shared that CHWs were able to reliably screen clients with health problems at home or within the community, and link them to facility-based primary care. This work decreased barriers to diagnosis and treatment, alleviated pressure on the facility-based primary care workers, and decreased client wait time:

‘These people are helpful. They help in minimising waiting time. It doesn’t take as the CHW also indicates the problem on the referral form …’ (P10, female, healthcare provider, facility 4)

‘They also help to bring patients quickly to the facility. Because of the good work in patient identification, they have minimised referral cases here. We deal with patients successfully as they come in good time … they have minimized pressure at the referral facility.’ (P12, male, healthcare provider, facility 3)

Following a medical diagnosis, participants shared that the CHWs provide counselling to patients on disease processes, medication adherence, and the need for regular follow-up with appointment reminders. Participants agreed that CHWs decreased physical and social barriers in shifting several important tasks to the household and community level from the facility-based primary care workers and serve as a trust advisor:

‘… [*I*]f we did not have CHWs working on the ground, people could have died … Some do not even know that they are sick. But because the CHW assesses them, they are linked quickly to the facility and put on medication.’ (P6, male, healthcare provider, facility 4)

‘Previously we had many defaulters and even the new clients were not taking medication … CHWs have helped counselling the clients to understand that being HIV-positive does not mean that they are going to die. They even visit them daily to check medication adherence and remind them of appointment dates and escort them to the facility.’ (P9, female healthcare provider, facility 4)

The participants shared stories about the critical trust between CHWs and facility-based primary care workers facilitating patients to seek and receive care. Facility-based direct care workers conveyed that they were ready to share patient information with CHWs, with patient consent to ensure high-quality care and create opportunities for CHWs to further engage with the community. Likewise, community members trusted their CHWs with personal health information and regarded them as their ‘doctors’:

‘… [*S*]ometimes, with consent from clients, we share even confidential information on clients that helps CHWs as they provide continued support to such clients in the community.’ (P10, male, healthcare provider, facility 2)

‘I for one, I trust my CHW. He is my personal doctor such that I cannot hide anything from him. Hiding is a great mistake. Unless I want to die … If we open up with them, it becomes easy for them to offer the necessary help.’ (P3, female, community member, facility 3)

Even though CHWs can offer community-based care, they often travel long distances on foot in rural areas to reach households with difficulty reaching all assigned homes. With these transport difficulties, participants reported it was challenging for patients to receive transport, especially when they were very sick. Often CHWs provided money or physically carried individuals to health facilities. Participants suggested the need for additional transport support:

‘They should also be provided with bicycles to ease their work as they travel long distances visiting households and escorting clients to the health facility.’ (P5, female, community member, facility 3)

‘The first challenge is transporting. Sometimes they find a client in the community who is too sick and can’t walk. It is the CHW who will try all means making sure that the client has been taken to the health facility even though they receive little money.’ (P2, male, community member, facility 4)

### Motivation (‘the why’): The community health worker programme enablers and challenges in providing community-based primary health care

Community health workers enable community-based primary care through increased social interactions which increases trust among community members, CHWs, and facility-based primary care workers, promoting PHC and linking communities to health facilities:

‘Our CHWs have strengthened the relationship between chiefs and their people as they link them through different forums. CHWs encourage us to love each other at all times. CHWs have helped us to coordinate with our neighbors when someone is sick, and we go to the health facility as a team.’ (P1, female, community member, facility 3)

‘CHWs are a very trusted cadre. We rely on them to link us to clients since we are understaffed. They are [*the*] eyes, ears, hands, legs, and a voice of those that work at the facility level; hence we trust them.’ (P6, male, healthcare provider, facility 1)

Motivational challenges within the CHW programme included workload, client outreach and community religious or spiritual beliefs. The prohibitive workload was often noted due to the high numbers of households assigned to the CHWs, which often exceeded the capacity of individual CHWs:

‘Some CHWs have 50 households … it is not just teaching, you have heard of accompaniment also. That means when they come to the facility, home visits are put on hold. Also they have children to take care of … would be reasonable if they can be given 15 to 20 households. They fail to manage their children because they are always up and down …’ (P6, male, healthcare provider, facility 2)

Participants expressed community motivation challenges including full participation of some individual groups in CHW activities, limiting their tasks. Participants noted that often adult men are not available for the CHW household visits and subsequently miss screening, health education, and potential referral.Even when the CHW booked an appointment with a particular household, a man was often still absent:

‘When CHWs are visiting households, mostly they do not find men at home. This is a huge challenge for us here because some of the teachings would be better if men heard them, but they always give excuses even when the CHW books them in advance.’ (P3, female, community member, facility 3)

Participants reported that religious and traditional beliefs within the community could also be a barrier to health promotion by CHWs. Some churches and religious groups believe that sick patients will heal with prayers. These institutions may encourage patients to stop taking medication, while others have deep trust and belief in traditional medicine. These actions challenge CHW’s tasks of screening, psychosocial and medication support:

‘… [*A*] client in our location went to a pastor and could not take medicine when fasting. She became very sick. The CHW talked to her … and she is now fine.’ (P2, male, community member, facility 3)

‘… [*T*]here are some illnesses like epilepsy that cannot be cured at the hospital but witch doctors. It is an issue of evil spirits …’ (P8, male, community member, facility 2)

## Discussion

Participants identified crucial roles for CHWs in communities, including enabling health knowledge, increasing care-seeking behaviours, building trust and social connections, and improving access to the PHC health system. Following the COM-B framework (Figure 1), CHWs enhance health behaviour change with increasing capacity, opportunity, and motivation to facilitate the delivery of PHC.

Through direct placement in the community, CHWs promote holistic health care for individual clients and the community, thereby decreasing stigma and discrimination. For example, participants shared that CHWs influenced hygienic practices through health promotion. Participants reported that community members were more apt to be screened and seek care for TB and other chronic diseases with CHW involvement. These results mirror results from South Africa, which demonstrated that CHWs working in PHC improved health knowledge and care-seeking activities.^1^ Furthermore, by increasing health knowledge and healthcare-seeking behaviours, CHWs facilitate feelings of well-being and social connections, as supported in other recent CHW programme studies.^8,41,42,43^ However, CHWs travel long distances on foot to reach households and sometimes do not have ways to help transport sick patients to the closest health facility. Previous studies have demonstrated that adequate scope, workload, and support are essential in empowering CHWs.^41,44,45,46,47^ Tsolekile et al.^47^ found that CHWs often perform tasks outside their training inhibiting their ability to offer high-quality, efficient services. Increasing support and resources can significantly enhance the effectiveness of CHWs, ensuring better access to quality healthcare for underserved communities.

Participants perceived CHWs as knowledgeable and capable of finding and supporting patients with new and existing health problems. Community health workers support facility-based PHC workers with finding patients through screening and referral, assisting in patient monitoring with perceived improvement in adherence, and minimising default rates studied and reported elsewhere.^41^ This CHW work decreases the workload for facility-based health care workers and provides home-based direct patient support.^48^ Similar findings have been reported in CHW programmes across sub-Saharan Africa.^1,10,11^ For example, a study in South Africa demonstrated that CHWs usually perform screening by nurses at the facility, thus alleviating workload and improving screening rates at health facilities.^1^ Community health worker roles at a household level help them promote, educate and discover illness, enabling PHC at the community level. Our findings, indicate that CHWs face programmatic and community challenges that demotivate them in completing their tasks, for example, a high number of assigned households. Participants suggested reducing the number of homes per CHW to increase productivity. Additionally, CHWs were reported to be limited by their capacity, lacking job aids and training to complete their tasks. Other studies in South Africa recommend frequent refresher training and supportive supervision for ongoing CHW improvement.^1,47^ We must ensure that CHWs, communities, and facility-based primary care workers collaboratively work to optimise CHW roles, scope, and geographical spread for a productive CHW workforce to provide high-quality services to rural populations.

Participants reported challenges in cultural and spiritual practices for CHWs to perform their tasks. Participants conveyed that religious beliefs can contradict the health messaging and promotion CHWs provide in the community. In Malawi, many individuals strongly believe that health problems originate from the spiritual world and that they can be treated with prayer and traditional medicines.^49,50^

Despite these beliefs, some participants shared that CHWs can often provide psychosocial support and education on health and treatment through their trusted roles and relationships. Musumari et al.^51^ reported religion and spiritual beliefs as facilitators and barriers to antiretroviral therapy (ART) adherence.^51^ Further studies must examine ways health care workers, including CHWs, can promote and combine spiritual and formal health practices to complement rather than oppose each other to benefit communities.

Finally, from a community perspective, participants reported poor male involvement in CHW household visits as a barrier to providing primary care. Men often work outside the household and do not prioritise CHW visits. This leads to a lack of completed screening or referrals, and health education. This decreases CHWs’ efficacy in two ways: (1) they are unable to provide health education and screening with encouraging care-seeking behaviours for men, perpetuating the cycle of poor care seeking, and (2) men are the primary decision-makers in the family, and their absence compromises the uptake of health-seeking behaviours for the entire household. These challenges have also been reported in West Africa^52^ where poor male involvement in family planning decision-making affected its uptake. Community health programmes and facility-based PHC must creatively find ways to promote dialogue and practices for increased male interaction with health care education, promotion, and treatment services.

### Limitations of the study

This study is limited in generalisability as it was completed in one remote district in Malawi. However, the Neno district’s terrain, economy, cultural beliefs, and health care systems are not unlike much of sub-Saharan Africa, making the findings widely applicable. widely applicable. Similarly, CHWs in Neno district focus on health education, screening, linkage, patient follow-up, and psychosocial support without diagnostics or medications. This may differ from other CHW programmes in Malawi or elsewhere, but many of the enabling resources and challenges have been reported in other PHC systems, regardless of specific services. Finally, the COM-B model has been criticised for omitting ‘wanting’ as a causal factor in behaviour change.^53^ However, in our interpretation of ‘motivation’, we have included both ‘need’ and ‘want’.

## Conclusion

Community health workers play a mediating and key supporting role between the health care system and vulnerable communities by being a trusted resource, providing health promotion, enabling healthy behaviours, and alleviating the workload for facility-based health care workers. Programmatic adjustment is recommended to improve the education of CHWs, decrease workload, and improve travel logistics for CHWs to enrich further and strengthen the PHC system. We recommend that CHW programmes, policymakers, and health care stakeholders work with CHWs and vulnerable communities to mitigate challenges and facilitate active partnerships and trusted linkages between communities and the PHC system.

## Appendix 1: Focus group discussion and individual interview guide

Perspectives of community members and health facility workers on the enablers and challenges of the community health worker role in community-based primary health care in rural Malawi

### Interview guide

**TABLE 1-A1:** Community Member Focus Group Questions (members of households who receive community health worker services, other Community Stakeholders).

Questions (and probes)	What the question measures
What are the roles that CHWs play in your community?	‘Icebreaker question’; Knowledge and beliefs about what CHWs provide
What are the ways that you think CHWs should support households and patients?	
How would communities be different if there were no CHWs working?	
When a CHW visits a household, in your personal experience, what does the CHW typically do during a visit?	How are CHWs spending their time; general patient perspectives on CHW services
Of these things you’ve mentioned, which ones take up the most time?	
Do you think the most helpful things take up the largest amount of time, or is that not always true? Why do you think it is like this?	
Is there anything you would like to change about what your CHW does, so that they could be more helpful to you?	
Currently, CHWs are trained to focus on key health priority areas (HIV, TB, maternal health including ANC, facility-based deliveries, post-natal care, under-5 malnutrition, STI and NCDs). Do you think these are the best things for them to focus on, or would you recommend any changes?	Acceptability of focus areas
How much interaction do you have with people in your extended family and community?	Social Connectedness
Do you feel your CHW has changed that in any way?	
Has your CHW helped you find resources or support in the community that you did not previously know existed?	
Do you feel that your relationship with your CHW has changed now that you have the same person for all of your families’ needs?	
Have you ever requested your CHW come to your house more than once a month to visit and why?	
What, if any, do you feel is a CHW’s effect on the larger community apart from the family they are assigned to?	
What are your views on CHWs’ capacity to address health issues that are assigned to them? issues?	Knowledge/Capacity of CHW
What issues are they most capable of addressing? Least capable?	
Can you give us an example of a time when you or someone you know asked for health advice from your CHW?	
How much do CHWs keep information confidential?	Perceptions of Trust
Would you be comfortable sharing personal health issues with a CHW? Why/why not?	
Can you give an example of when a CHW might help with issues in the households beyond what they are directly required to do?	
Does the CHW influence your decision about whether to go to a health facility, or when to go?	CHW influence on health care-seeking attitudes and behaviours
Why/why not?	
Can you think of any times when you or anyone you know went to the health facility specifically because of support from a CHW?	
*← If yes: probe with follow-up questions to get details about what the CHW did, why their actions helped, how things might have been different if the CHW was not there.*	
*← If no: are there different things the CHW could do to make it more likely that people will go to the health facility when they need care?*	
What barriers do community members face when they need to access health care at the facility?	Barriers to care and delays in seeking care
Can you think of any examples where CHWs helped reduce those barriers to health care for first time patients? Returning patients?	
Probe for examples	
What do people in the community think when they see a CHW going into someone’s house?	Positive or negative impressions of why a family might need CHW support (e.g. stigma)
Probe as needed to clarify why community members feel a certain way	
How do CHWs compare to other people that provide health-related services in the community, like HSAs or traditional healers?	Perceive differences between CHWs and other health cadres (e.g. HSAs)
In general, how do you think people in this community decide whether to seek help from a CHW or another person?	
If they seek care elsewhere, where might they go and why?	
Under what circumstances might someone prefer to get support from a CHW than an HSA?	
Under what circumstances might someone prefer to get support from an HSA than a CHW?	
Under what circumstances might someone prefer a traditional healer?	
Both men and women can serve as CHWs. In your opinion, is this appropriate?	Gender perceptions that may affect CHW programme implementation
Who has a CHW who is male? Who has a CHW who is female?	
For those who have a CHW that is female;	
Are there things best done by a female CHW?	
What happens when a male CHW does those things?	
For those who have a CHW that is male	
Are there things best done by a male CHW?	
What happens when a female CHW does those things?	
Do you think the gender of a CHW is important if they are supporting a pregnant woman? Or counselling around family planning?	
Do you think community members feel equally comfortable with a male or a female CHW?	
What is good about the services that CHWs offer in your village?	General perceptions; interview wrap-up question
Is there anything that you would change about how CHWs currently do their work?	
Are there some tasks that CHWs do that you think they should NOT be doing? What are these tasks?	
Are there tasks they do not currently do that you think they should start doing? What are these and why should they start doing them?	

CHW, community health worker; HIV, human immunodeficiency virus; TB, tuberculosis; STI, sexually transmitted disease; NCD, non-communicable disease; ANC, antenatal care; HSA, health surveillance assistants.

**TABLE 2-A1:** Healthcare Worker Focus Group Questions.

Questions (and probes)	What question measures
What roles do CHWs play in the community?	‘Icebreaker question’; also a good way to see whether CHWs independently mention anything relevant to social connectedness.
What are the ways that you think CHWs should support clients/households assigned to them?	
How would communities be different if there were no CHWs working?	
Let’s talk about what a typical day of work looks like for you. Can you walk me through the major activities that take up most of your time?	General understanding of how CHW work and how time is spent
Do CHWs help you do your work? How? (*Probe for specific examples)*	
Site Supervisors – How much time do you spend on supervising CHWs?	
Overall, what parts of the CHWs’ job do you think are the most helpful and effective at improving the health of the households they support?	CHW uses of time and possible changes to improve program
Are there any ways that their responsibilities could be changed to give them more time doing the things that are most helpful and important to their clients or the community?	
If yes, what would these changes be?	
Under the Household Model, CHWs are trained to focus on the following key health priority areas: HIV, TB, maternal health including ANC, facility-based deliveries, post-natal care, under-5 malnutrition, STI and NCDs.	Acceptability of focus areas
Do you think these are the best areas for them to be focusing on, or would you recommend any changes?	
Do you think CHWs have the capacity to address health issues that are assigned to them?	Knowledgeability/Capacity of CHW
What issues are they most capable of addressing? Least capable?	
Are CHW facilitating relationships beyond their scope of work with the community as well as household members?	Social connectedness
Have the CHWs been able to help patients/households find support in the community that they did not otherwise know about? If so, can you give me a specific example?	
(If the participant worked within the old model) Since switching over to the new model where CHWs are being assigned to specific households, have you seen the CHW relationship or your relationship with patients change at all? If so, how?	
What, if any, do you feel is a CHW’s effect on the larger community apart from the family they are assigned to?	
What (if any) behaviour changes have you noticed over time in the patients/households that CHWs work with?	CHW influence on health care-seeking attitudes and behaviours
Can you tell me about a specific time when a CHW helped a patient to do something that they would not have done without their support?	
Would you say that CHWs are trusted with confidential information in your community?	Perceptions of Trust
If yes, what makes you think so?	
If no, why not?	
In what ways are CHWs helping to address health conditions impacting your community?	Linkage to Care
Can you think of an example of when a CHW identified a person or household and linked them to the health facility that would not have otherwise gone for care?	
What are your interactions with the CHWs like?	Supervision and bi-directional communication
Is the current supervision structure effective?	
Based on your experience what are the major gaps that you have identified in how CHW are supervised and communicate with supervisors?	
Are there supports that CHWs are currently not getting that you think would be helpful for them in the future?	
Currently, one CHW is assigned to 20–40 households. Based on your experience, is that a feasible number of households to give to one CHW? Why?	Ideal CHW to household ratios, solutions for scale-up
What would be the highest number of houses a CHW could effectively support per day and why?	
What is the most ideal number and why?	
Both men and women can serve as CHWs. In your opinion, is this appropriate?	Gender perceptions that may affect CHW programme implementation
Do you think community members feel equally comfortable with a male or a female CHW?	
Are there things that are best done by a female CHW but not a male? Or things that are best done by males, not females?	
Do you think the gender of a CHW is important if they are supporting a pregnant woman? Or counselling around family planning?	
As you probably know, recently APZU updated the CHW strategy to focus on households rather than individual patients.	General perceptions; interview wrap-up question
Overall, what has been your experience now that APZU is using this household model for CHWs?	
If you worked in Neno before the household model came into place, what are the main differences in your work comparing now to the time before household model?	
What do you like most about working within the household model?	
What are your main challenges with the household model?	

CHW, community health worker; HIV, human immunodeficiency virus; TB, tuberculosis; STI, sexually transmitted disease; ANC, antenatal care; NCD, non-communicable disease; APZU, Abwenzi Pa Za Umoyo.
